# Supplementary figures and images for: Structures of proteinase 3 and the CD177 receptor complex reveal a major autoantibody epitope
Source: EMBO Rep. 2026 Feb 17;27(6):1580–606. doi: 10.1038/s44319-026-00716-5 (PMC13022285; doi:10.1038/s44319-026-00716-5)

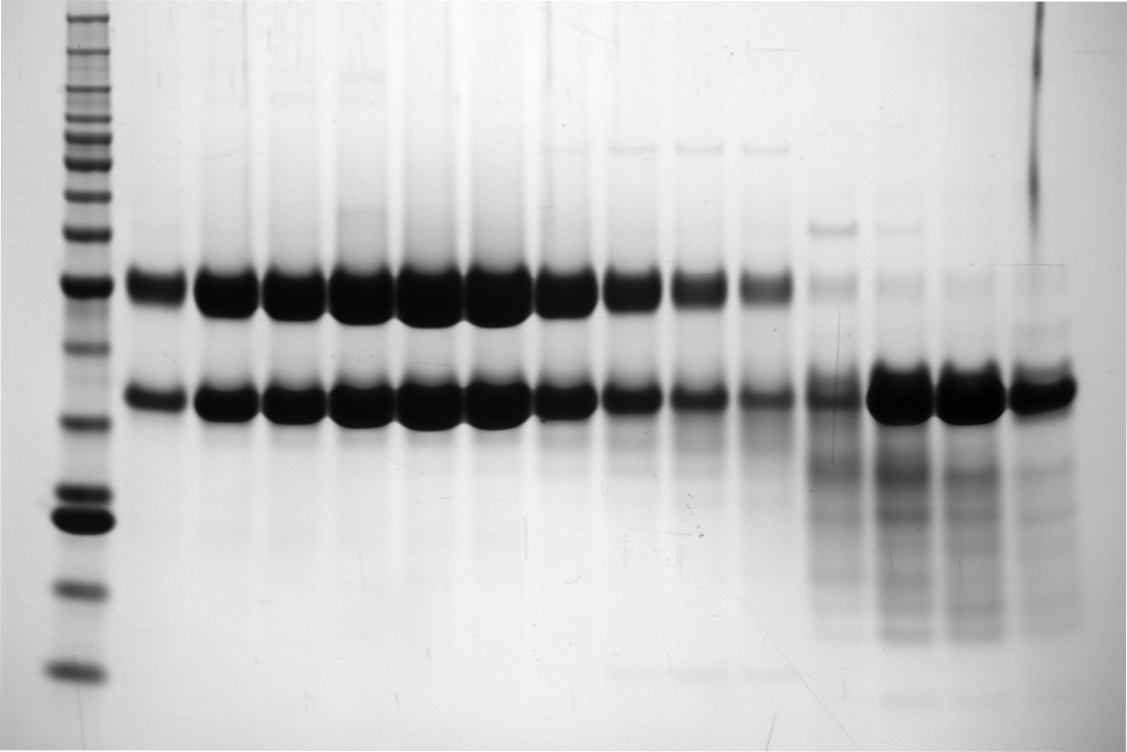

Supplement: Supplementary file 7 — Figure EV Source Data [file 44319_2026_716_MOESM7_ESM.zip › EV_figures/EV2B/PR3rec-CD177 complex purification.png]

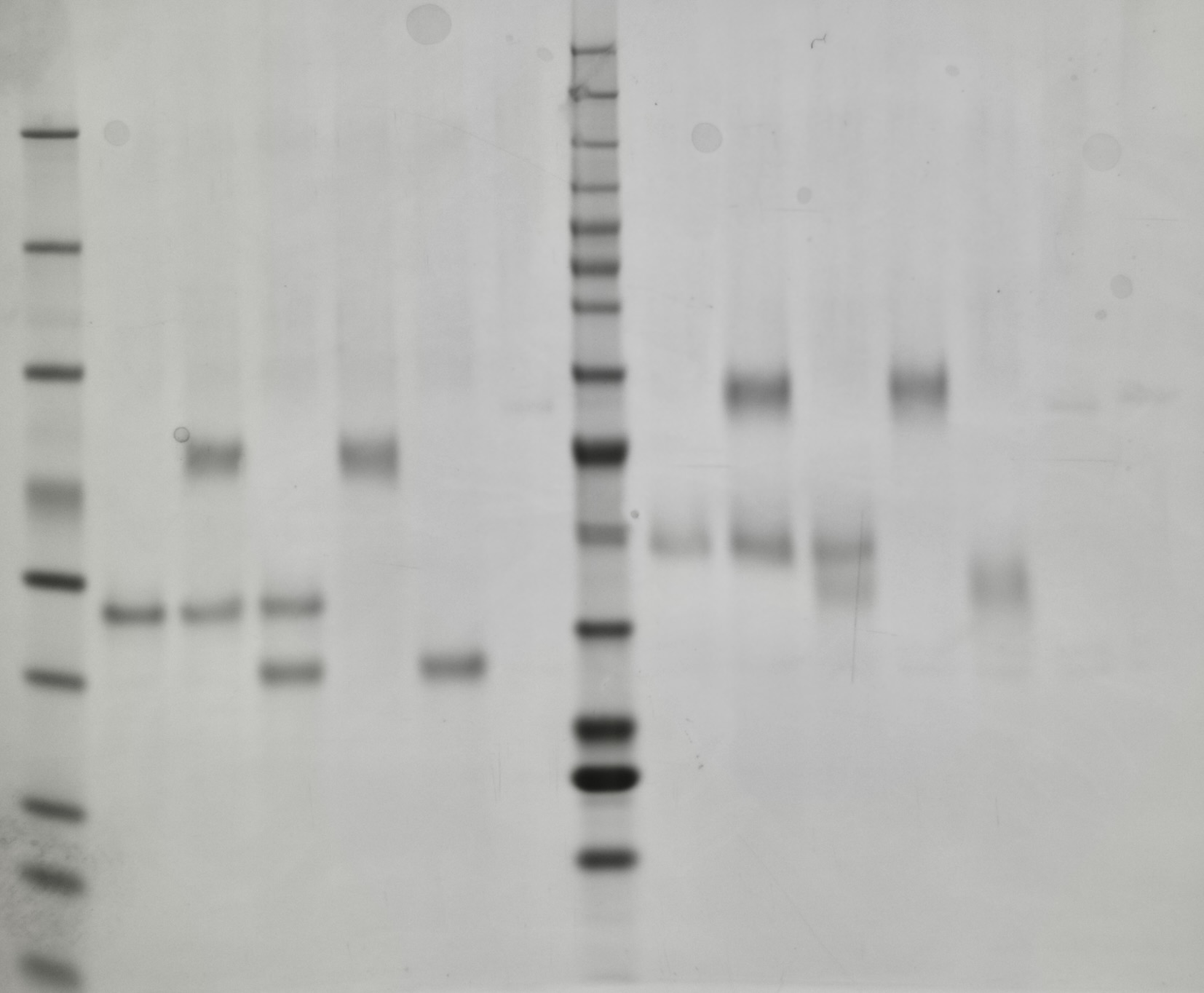

Supplement: Supplementary file 7 — Figure EV Source Data [file 44319_2026_716_MOESM7_ESM.zip › EV_figures/EV2D+E/SDS-PAGE gel of all constructs.jpeg]

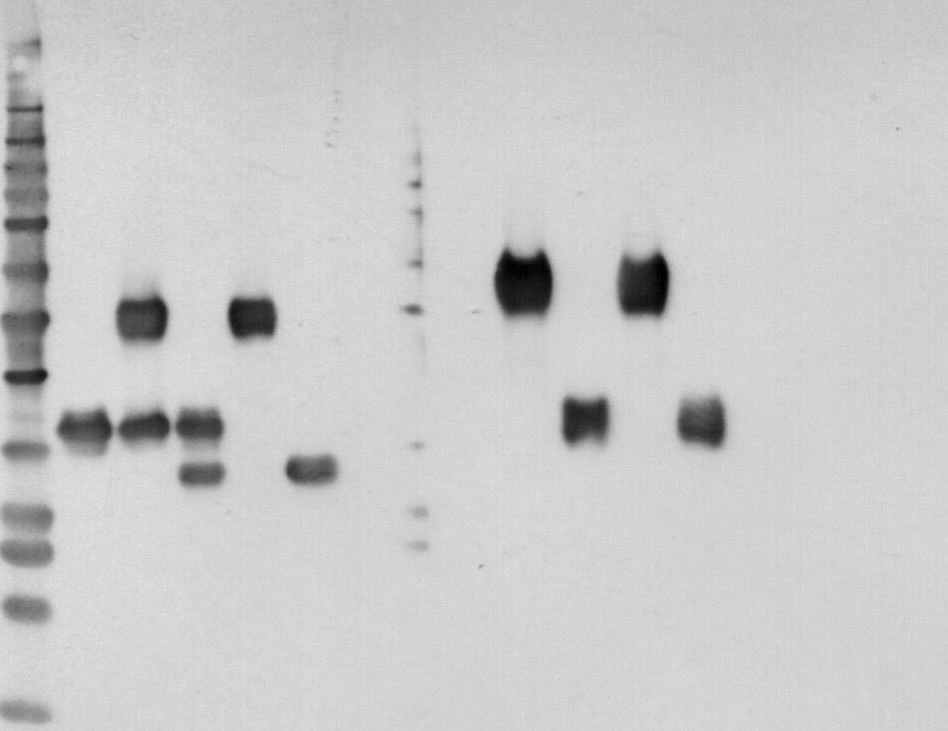

Supplement: Supplementary file 7 — Figure EV Source Data [file 44319_2026_716_MOESM7_ESM.zip › EV_figures/EV2D+E/a-his and a-strep western blot of all constructs.jpg]

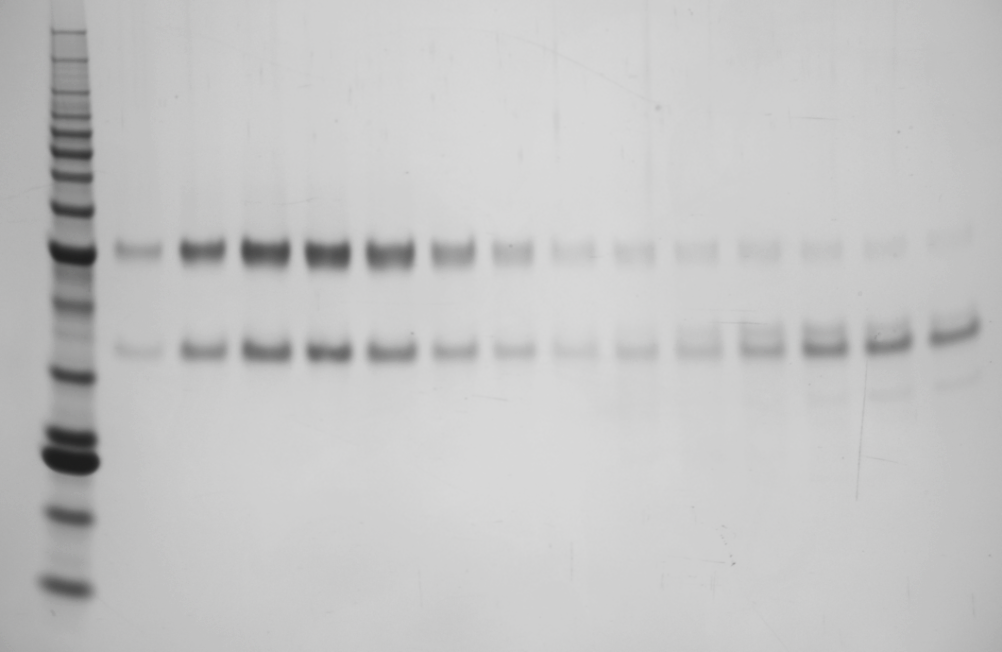

Supplement: Supplementary file 7 — Figure EV Source Data [file 44319_2026_716_MOESM7_ESM.zip › EV_figures/EV1I/PR3rec-CD177 SEC binding assay.png]

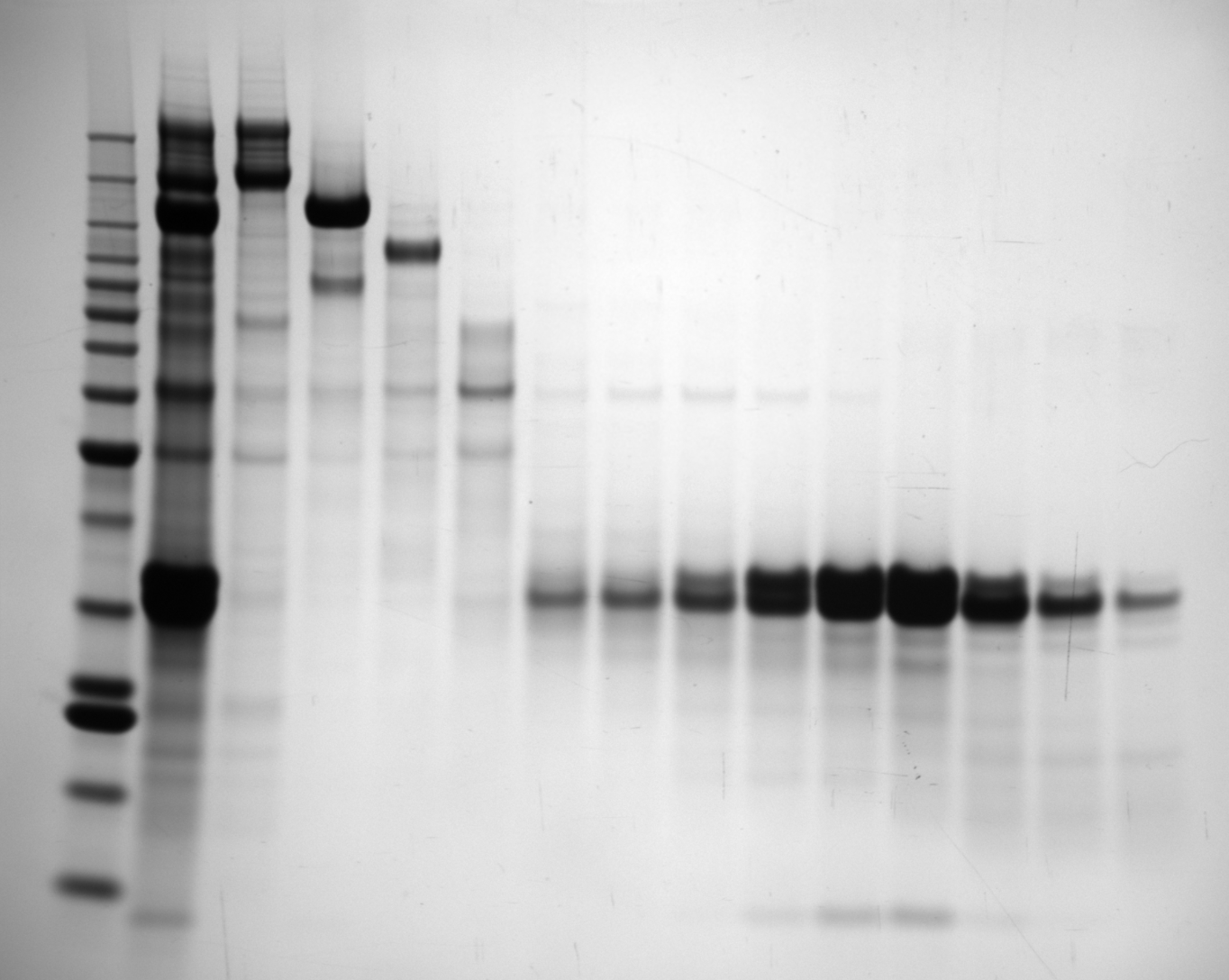

Supplement: Supplementary file 7 — Figure EV Source Data [file 44319_2026_716_MOESM7_ESM.zip › EV_figures/EV1G/PR3rec purification SDS-PAGE gel.png]

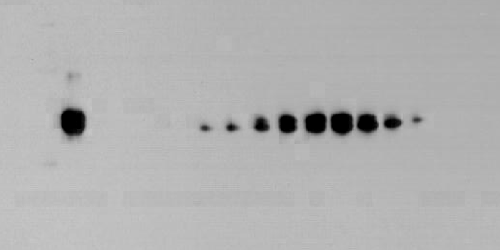

Supplement: Supplementary file 7 — Figure EV Source Data [file 44319_2026_716_MOESM7_ESM.zip › EV_figures/EV1G/PR3rec purification anti-his blot.png]

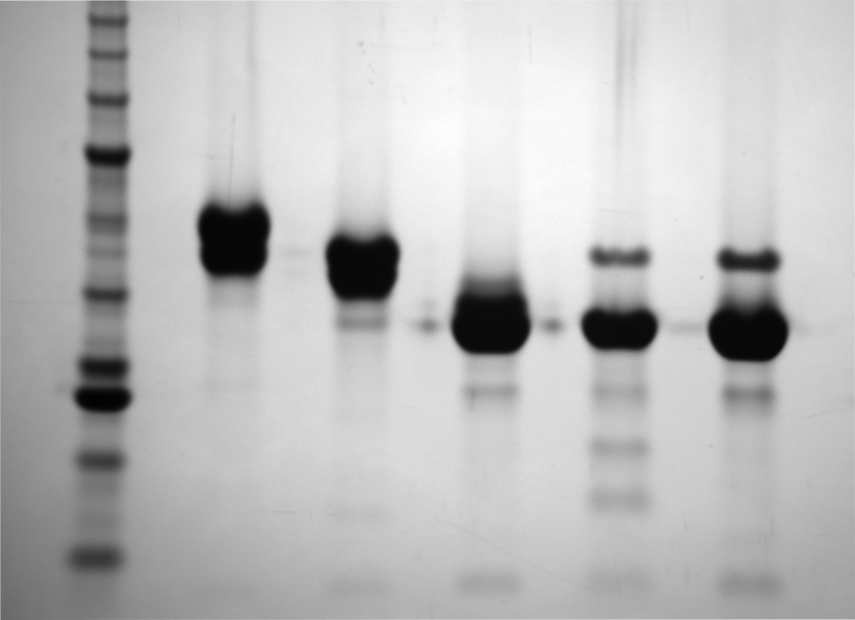

Supplement: Supplementary file 7 — Figure EV Source Data [file 44319_2026_716_MOESM7_ESM.zip › EV_figures/EV1H/Deglycosylation assay.png]

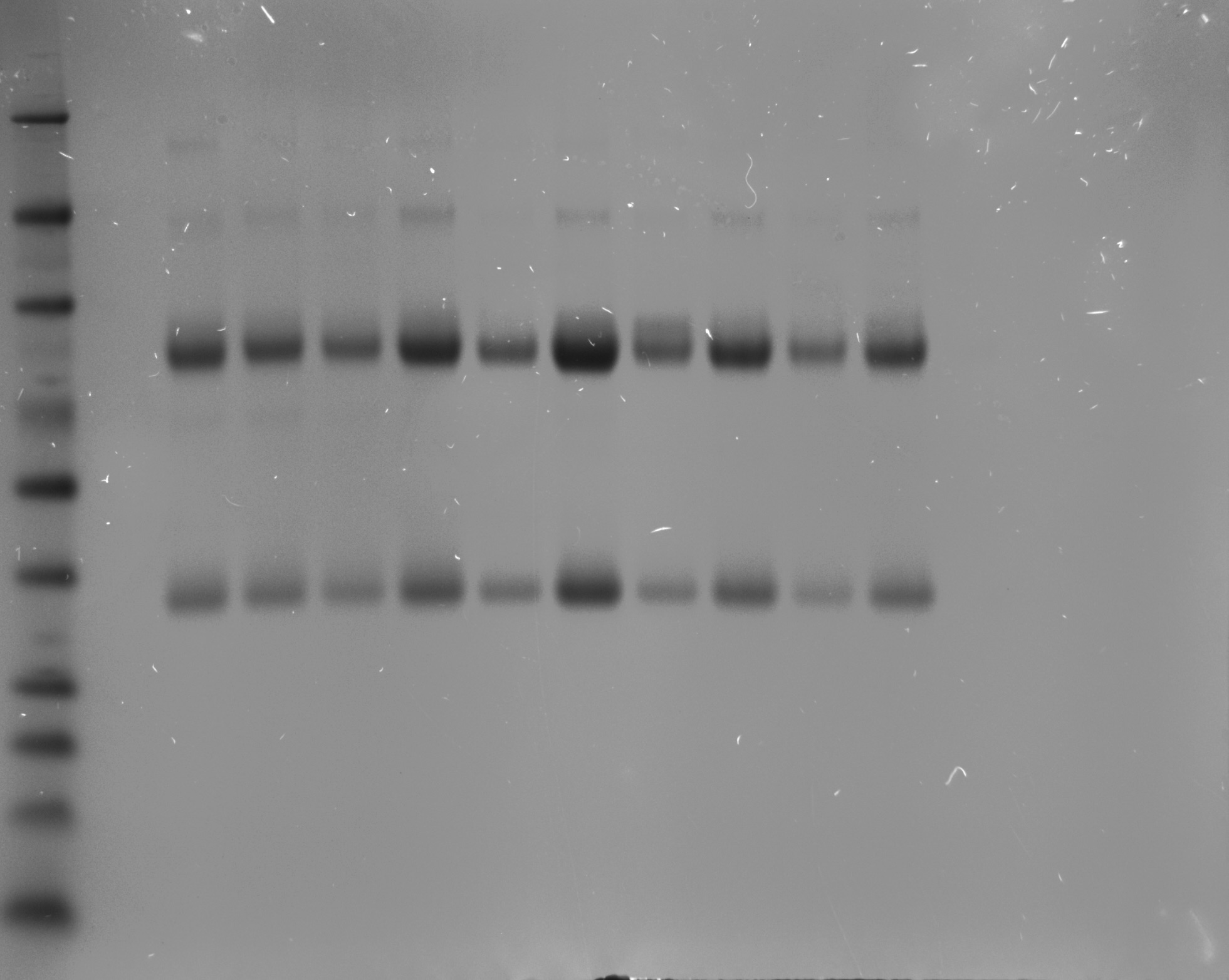

Supplement: Supplementary file 7 — Figure EV Source Data [file 44319_2026_716_MOESM7_ESM.zip › EV_figures/EV5A/SDS-PAGE of purified IgGs.jpeg]
