# Supplementary material for: Structures of proteinase 3 and the CD177 receptor complex reveal a major autoantibody epitope
Source: EMBO Rep. 2026 Feb 17;27(6):1580–606. doi: 10.1038/s44319-026-00716-5 (PMC13022285; doi:10.1038/s44319-026-00716-5)
Supplement: Supplementary file 8 — Expanded View Figures [file 44319_2026_716_MOESM8_ESM.pdf]

Expanded View Figures

Figure EV1. Structural and biochemical analysis.

(**A**) Sequence alignment of LU1-4 domains of CD177 (D1-D4) and LU domains from other proteins as indicated. The positions of predicted β-strands and disulphide bridges are indicated. (**B**) Plots of sulphur anomalous signal metrics as a function of resolution obtained from XSCALE and SHELXC softwares. SigAno represents the estimated anomalous signal strength while <d"/sig> shows the normalised difference signal. See Dataset EV3. (**C**) Structural superposition of the two copies of CD177^{ecto} found in the asymmetric unit of the crystal, shown in grey and cyan. (**D**) Sequence alignment of different PR3 orthologs of the hydrophobic (blue) and positively charged (orange) residues. (**E**) Cartoon representation of PR3 (PDB ID: 1FUJ). The catalytic triad, hydrophobic and positively charged residues in the hydrophobic surface patch are indicated by purple, blue and orange spheres, respectively. (**F**) Close-up view of mutated residues in the hydrophobic surface patch. (**G**) Data from recombinant PR3^{rec} protein purification from HEK293 cultures. The Size-Exclusion Chromatography (SEC) purification profile is shown as well as its corresponding Coomassie-stained SDS-PAGE gel (top) and anti-His western blot (bottom) with the eluted fractions. The main protein peak is indicated by a red asterisk. Void volume fractions are indicated in grey. Impurity fractions are indicated by yellow, orange and red arrowheads. Presence of PR3 (~ 30 kDa) in the analysed samples is indicated by a black arrow. (**H**) SDS-PAGE gel of glycosylated PR3^{rec} samples produced by HEK293T and N-acetylglucosaminyltransferase I-deficient HEK293S cells displaying the difference in glycosylation pattern and size between the two cell lines (lanes 1 and 2). Samples obtained after deglycosylation by the endoglycosidases Endo-F1 (lane 3) or PNgase F (lanes 4 and 5) confirm that PR3^{rec} is indeed glycosylated. (**I**) SEC-binding profile and corresponding SDS-PAGE gel of PR3^{rec} with CD177^{ecto}. PR3^{rec} and CD177^{ecto} profiles are shown in teal and grey, respectively. The profile of a PR3^{rec}-CD177^{ecto} protein mixture in a 2:1 molar ratio is shown in purple. The protein peaks are indicated by a purple asterisk. Source data are available online for this figure.

© The Author(s)

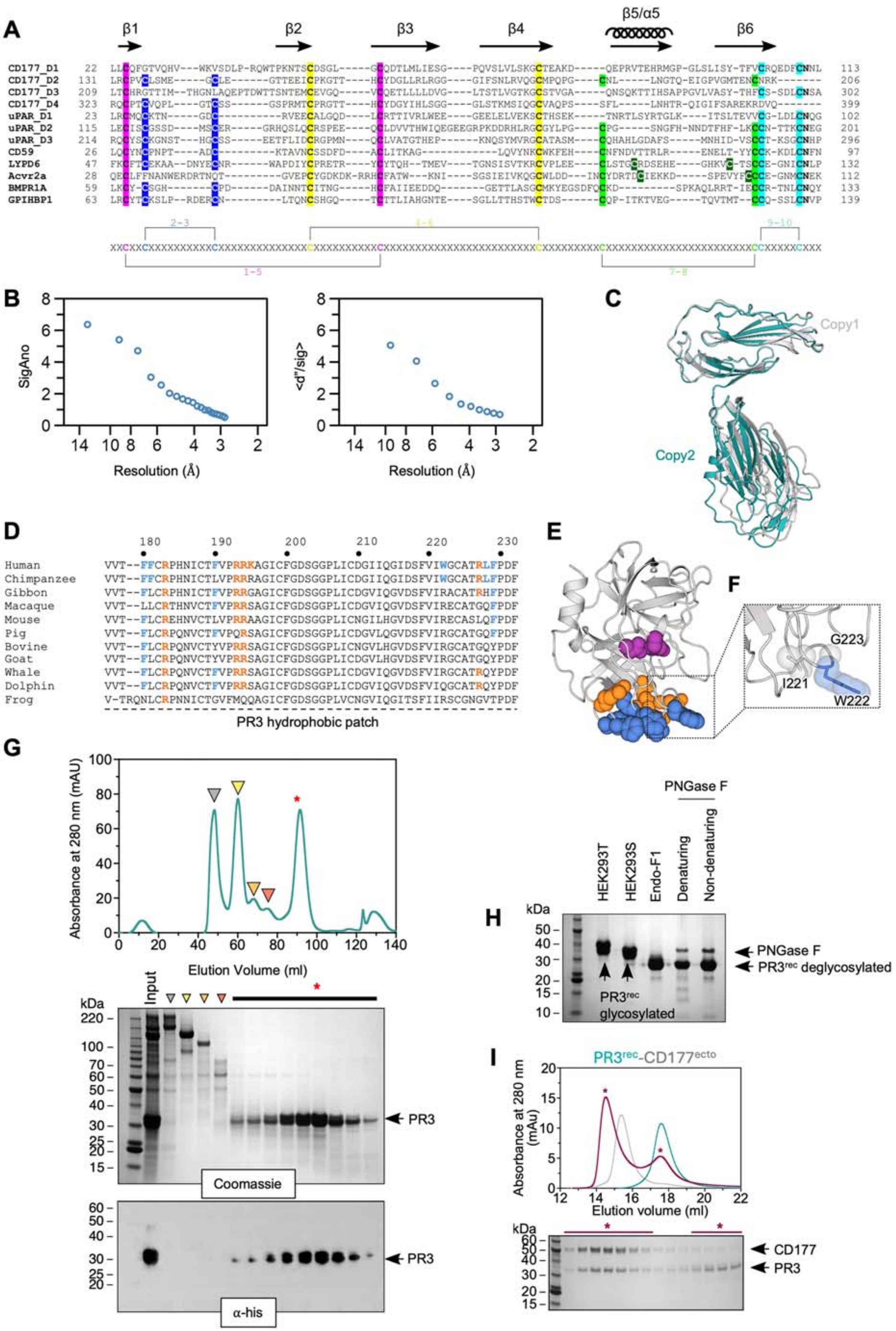
A
β1
β2
β3
β4
β5/α5
β6
CD177_D1
CD177_D2
CD177_D3
CD177_D4
uPAR_D1
uPAR_D2
uPAR_D3
CD59
LYPD6
Acvr2a
BMPR1A
GPIHBP1
2-3
4-6
9-10
1-5
7-8
B
SigAno
Resolution (Å)
<d"/sig>
Resolution (Å)
C
Copy1
Copy2
D
Human
Chimpanzee
Gibbon
Macaque
Mouse
Pig
Bovine
Goat
Whale
Dolphin
Frog
PR3 hydrophobic patch
E
F
G223
I221
W222
G
Absorbance at 280 nm (mAU)
Elution Volume (ml)
kDa
Input
PR3
Coomassie
α-his
H
PNGase F
HEK293T
HEK293S
Endo-F1
Denaturing
Non-denaturing
PNGase F
PR3rec deglycosylated
PR3rec glycosylated
I
PR3rec-CD177ecto
Absorbance at 280 nm (mAu)
Elution volume (ml)
CD177
PR3

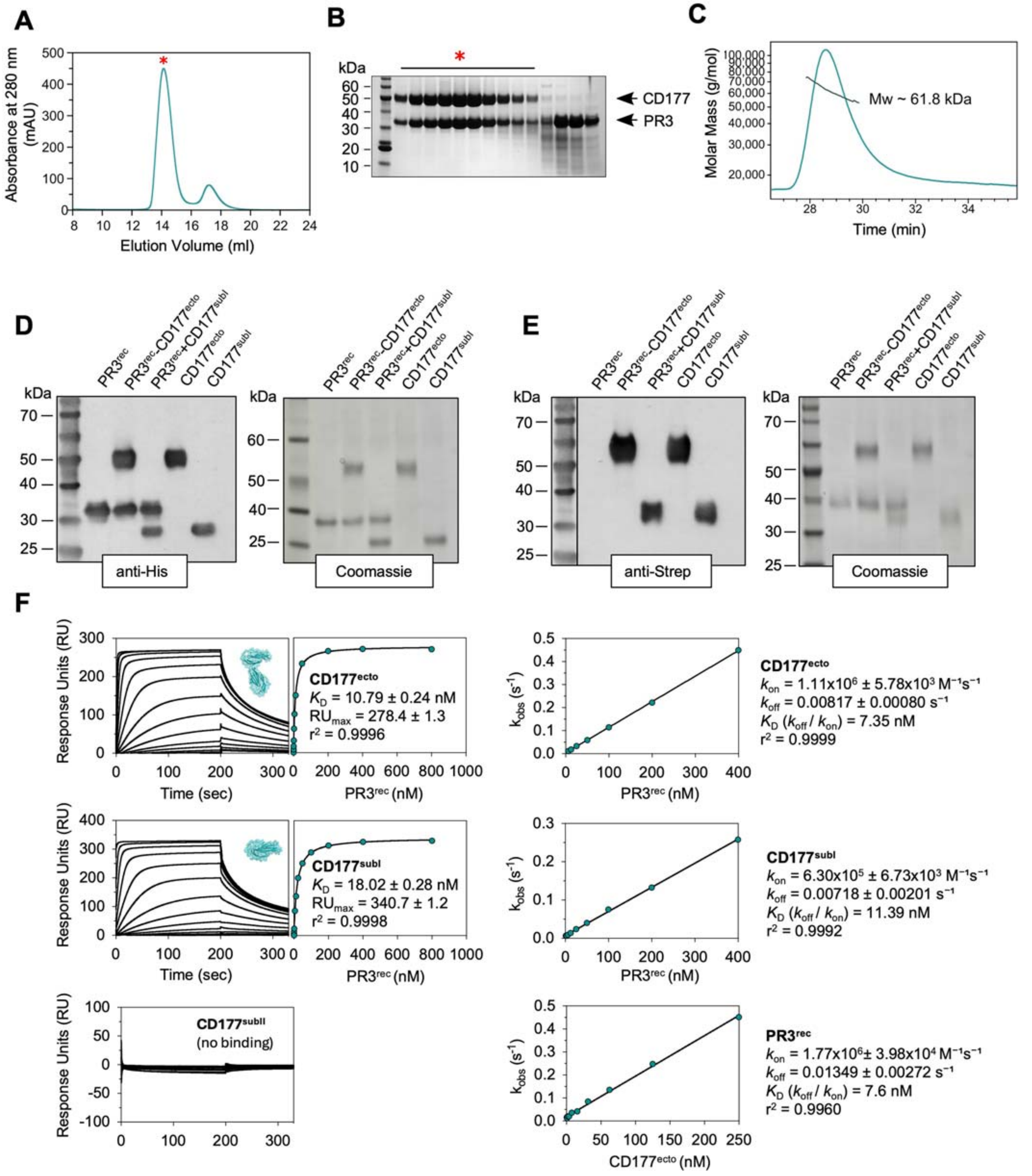
A
Absorbance at 280 nm (mAU)
Elution Volume (ml)
B
kDa
CD177
PR3
C
Molar Mass (g/mol)
Mw ~ 61.8 kDa
Time (min)
D
PR3rec
PR3rec-CD177ecto
PR3rec+CD177subI
CD177ecto
CD177subI
kDa
anti-His
Coomassie
E
anti-Strep
Coomassie
F
Response Units (RU)
Time (sec)
PR3rec (nM)
CD177ecto
KD = 10.79 ± 0.24 nM
RUmax = 278.4 ± 1.3
r2 = 0.9996
kobs (s-1)
CD177ecto
kon = 1.11x106 ± 5.78x103 M-1s-1
koff = 0.00817 ± 0.00080 s-1
KD (koff / kon) = 7.35 nM
r2 = 0.9999
CD177subI
KD = 18.02 ± 0.28 nM
RUmax = 340.7 ± 1.2
r2 = 0.9998
CD177subI
kon = 6.30x105 ± 6.73x103 M-1s-1
koff = 0.00718 ± 0.00201 s-1
KD (koff / kon) = 11.39 nM
r2 = 0.9992
CD177subII
(no binding)
PR3rec
kon = 1.77x106± 3.98x104 M-1s-1
koff = 0.01349 ± 0.00272 s-1
KD (koff / kon) = 7.6 nM
r2 = 0.9960
CD177ecto (nM)

 © The Author(s)

Figure EV2. Purification and biophysical characterisation of $PR3^{rec}$-$CD177^{ecto}$ complex.

(**A**) SEC profile of purified $PR3^{rec}$-$CD177^{ecto}$ complex. The main protein peak is indicated by a red asterisk. (**B**) Coomassie-stained SDS-PAGE gel showing fractions eluted from SEC (experiment in (**A**)). (**C**) Multiangle light scattering experiment of $PR3^{rec}$-$CD177^{ecto}$ confirms a 1:1 interaction in solution, with the expected size of ~60 kDa for the complex. (**D**) anti-His western blot (left) and reducing Coomassie-stained SDS-PAGE gel (right) of His-tagged PR3 and CD177 constructs. Same sample preps were used for both experiments. The $PR3^{rec}$-$CD177^{ecto}$ sample comes from the SEC-purified complex peak as shown in panel A. The $PR3^{rec}$+$CD177^{subl}$ sample comes from mixing the two proteins together in a 1:1 molar ratio. (**E**) anti-Strep western blot (left) and reducing Coomassie-stained SDS-PAGE gel (right) of His-tagged $PR3^{rec}$ and TwinStrep-tagged CD177 constructs. Same sample preps were used for both gels. A different batch of $PR3^{rec}$ was used for these blots. (**F**) SPR data (left panels) and corresponding fitted curves (centre and right panels) show the binding of Avi-tagged $CD177^{ecto}$, $CD177^{subl}$ and $CD177^{subll}$ ligands (0.05 to 800 nM) to $PR3^{rec}$ analyte, and of Avi-tagged $PR3^{rec}$ ligand (0.06 to 1000 nM) to $CD177^{ecto}$ analyte (see sensorgrams in Fig. 3B). The immobilised levels were 560, 340, 320 and 350 resonance units (RU) respectively. For $PR3^{rec}$ binding to $CD177^{ecto}$ and $CD177^{subl}$ ligand, K_D were derived using both 1:1 equilibrium analysis (centre panel) and kinetic analysis (right panel). For $CD177^{ecto}$ binding to $PR3^{rec}$ ligand, kinetic analysis was performed with a 1:1 Langmuir model at concentrations below 16 nM, where binding curves reached clear plateau. At higher concentrations, an alternative model accounting for mass transport limitations was used. Final K_D values were determined from k_{ON} and k_{OFF} rates obtained independently from fits to association and dissociation phases. For SPR data, please refer to Dataset EV2. Source data are available online for this figure.

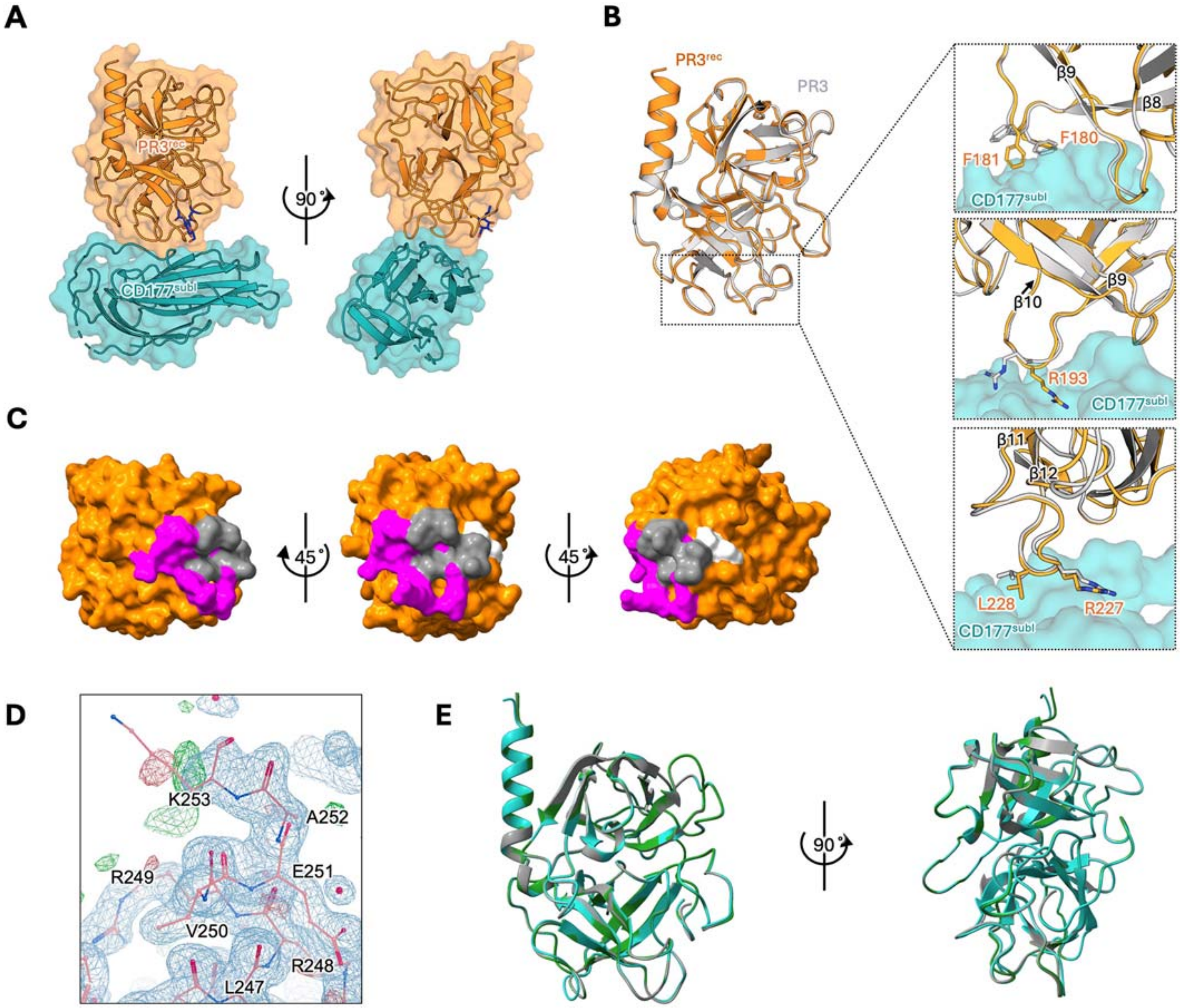

Figure EV3. Structural views of PR3^{rec} and PR3^{rec}-CD177^{subl}.

(**A**) Views of the PR3^{rec}-CD177^{subl} complex structure. (**B**) Superposition of the PR3^{rec} structure as found in complex with CD177^{subl}, and the previously published structure of PR3 (PDB: 1FUJ). Insets display close-ups on diverging side chains between PR3^{rec} and 1FUJ in the CD177-binding loops. (**C**) Views of PR3^{rec} with the CD177-binding region highlighted. Residues H148, G149, Q151, T176, V178, P192, R193, R227 and P230 are highlighted in magenta, residues I221N and W222G are highlighted in white and residues that overlap with the hydrophobic patch and CD177-binding region (F180, F181, F190, L228 and F229) are highlighted in dark grey. (**D**) The electron density maps calculated from data collected for the complex in panel A (2Fo-Fc at 1 sigma level in blue, Fo-Fc at $-/+$ 3 sigma level in green/red, respectively. The view is focused on PR3 K253. Protein model shown as sticks. (**E**) Superposition of AlphaFold predicted nPR3, PR3^{rec} and PR3^{nonCD177} structures. Grey: nPR3, Green: PR3^{rec}, Teal: PR3^{nonCD177}.

 © The Author(s)

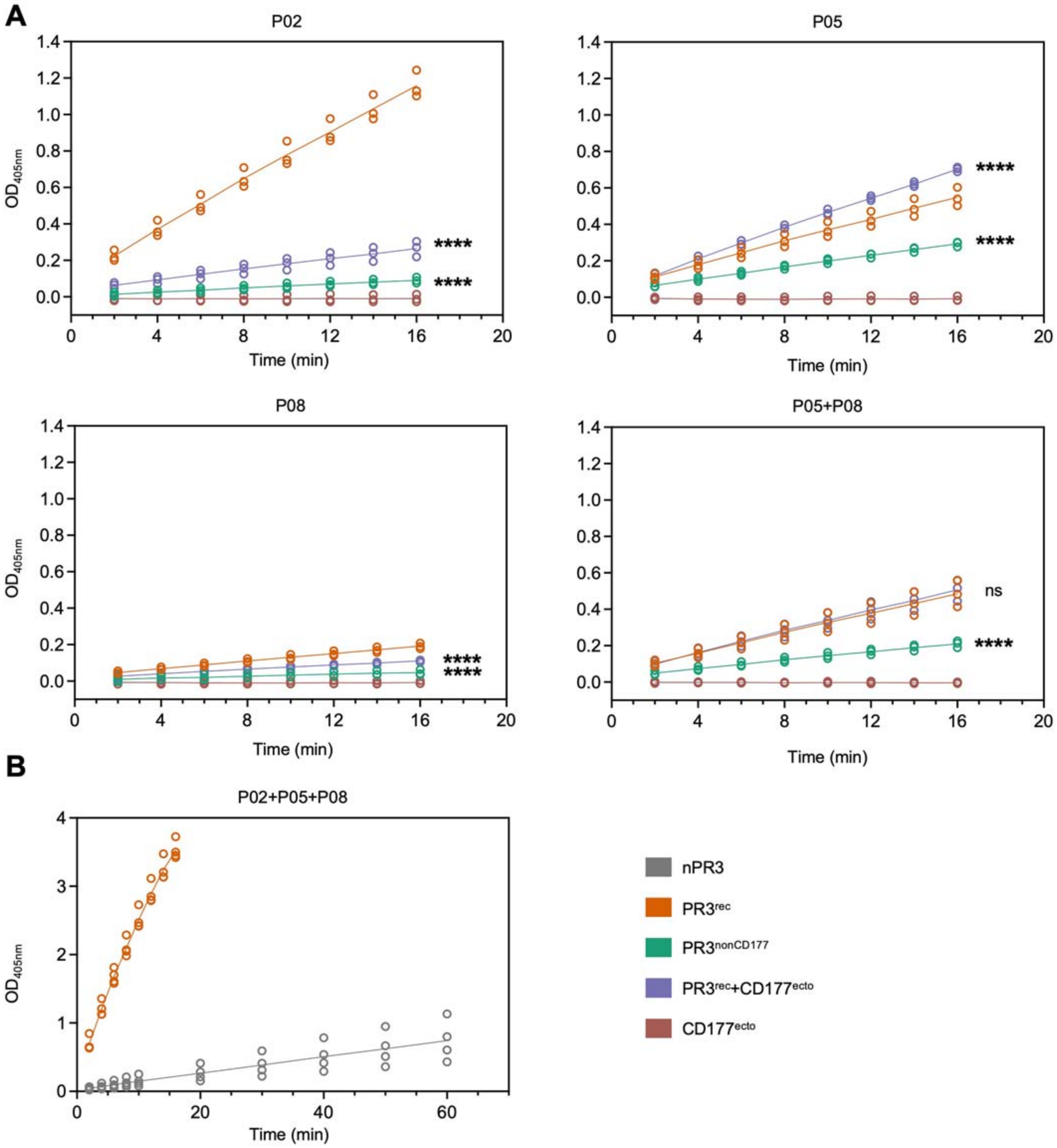

Figure EV4. Time-course ELISAs for individual plasma and plasma pool.

(**A**) Equal amounts of $PR3^{rec}$, $PR3^{nonCD177}$, $PR3^{rec}$+$CD177^{ecto}$ and $CD177^{ecto}$ were coated on ELISA plates for time course measurements using individual and mixed GPA patient plasma at a 1:1600 dilution. Simple linear regression analysis was carried out. Binding of $PR3^{nonCD177}$ and $PR3^{rec}$+$CD177^{ecto}$ were compared to $PR3^{rec}$ binding. $n = 3$. (**B**) Equal amounts of nPR3 and $PR3^{rec}$ were coated on ELISA plates for time course measurements using GPA patient plasma mixed in equal amounts (P02 + P05 + P08) at a 1:50 dilution. The results suggest that $PR3^{rec}$ offers faster detection of robust signal compared to nPR3. $n = 3$. *$P \leq 0.05$, **$P \leq 0.01$, ***$P \leq 0.001$, ****$P \leq 0.0001$. Data are presented as mean ± S.E.M. For exact P values, please refer to Dataset EV1. Source data are available online for this figure.

© The Author(s)

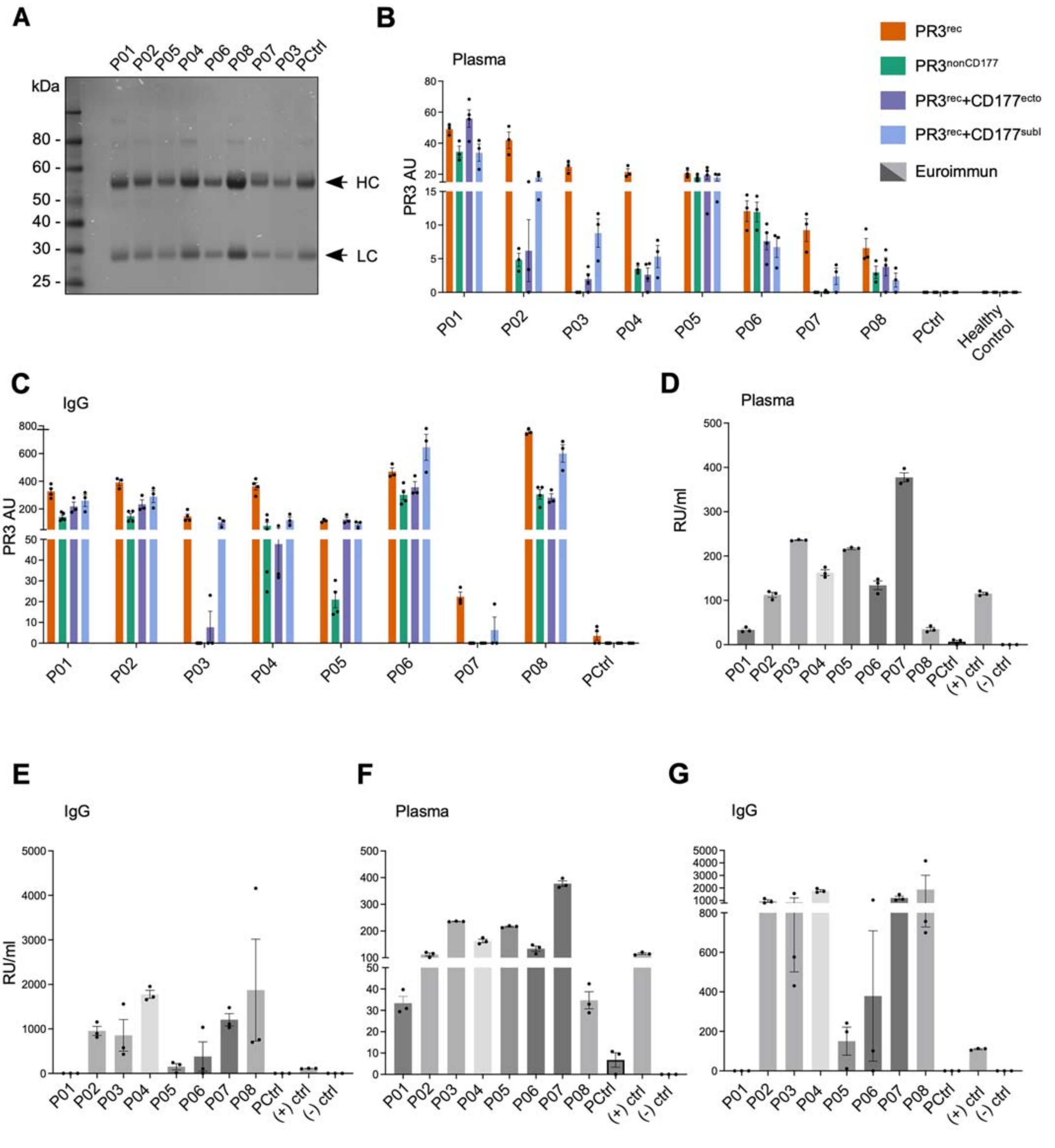
A
P01 P02 P05 P04 P06 P08 P07 P03 PCtrl
kDa
80
60
50
40
30
25
HC
LC
B
Plasma
PR3 AU
PR3rec
PR3nonCD177
PR3rec+CD177ecto
PR3rec+CD177subl
Euroimmun
P01 P02 P03 P04 P05 P06 P07 P08 PCtrl Healthy Control
C
IgG
PR3 AU
P01 P02 P03 P04 P05 P06 P07 P08 PCtrl
D
Plasma
RU/ml
P01 P02 P03 P04 P05 P06 P07 P08 PCtrl (+) ctrl (-) ctrl
E
IgG
RU/ml
F
Plasma
G
IgG

Figure EV5. IgG preparations from patient plasma.

(**A**) Reducing Coomassie-stained SDS-PAGE gel of patient purified IgG samples. Heavy chain (HC) and light chain (LC) bands are shown. Samples were loaded at 5 µg per well. (**B**, **C**) Zoomed in view of standardised ELISA data using plasma and IgG samples from PR3-ANCA positive GPA patients (P01-P08) and a MPO-ANCA positive patient (PCtrl), and plasma from a healthy control plasma sample. ANCA binding is reported in arbitrary units (PR3 AU). IgG samples were tested at 10 or 5 µg/mL. Responses were adjusted by the experiment dilution factor and then normalised by their purification dilution factor. $n = 3/4$. (**D**, **E**) Standardised ELISA data from a commercially available ELISA kit (Euroimmun) using the same patient samples. Binding is reported as response units per ml (RU/mL). (+) and (−) ctrl refer to the positive and negative controls provided with the kit. IgG samples were tested at 5 µg/mL and their purification dilution factor was used to normalise responses. $n = 3$. (**F**, **G**) Zoomed in views of (**D**, **E**). A minimum of three technical repeats were done for each ligand and sample. Source data are available online for this figure. $n = 3$. *$P \leq 0.05$, **$P \leq 0.01$, ***$P \leq 0.001$, ****$P \leq 0.0001$. Data are presented as mean ± S.E.M. For exact *P* values, please refer to Dataset EV1. Source data are available online for this figure.

© The Author(s)
